# Bite Injuries of Grey Seals (*Halichoerus grypus*) on Harbour Porpoises (*Phocoena phocoena*)

**DOI:** 10.1371/journal.pone.0108993

**Published:** 2014-12-02

**Authors:** Thierry Jauniaux, Mutien-Marie Garigliany, Pauline Loos, Jean-Luc Bourgain, Thibaut Bouveroux, Freddy Coignoul, Jan Haelters, Jacky Karpouzopoulos, Sylvain Pezeril, Daniel Desmecht

**Affiliations:** 1 Department of Veterinary Pathology, Fundamental and Applied Research for Animals & Health (FARAH), University of Liege, Sart Tilman B43, B-4000, Liege, Belgium; 2 Coordination Mammalogique du Nord de la France, 806 rue Haute, 62850, Alembon, France; 3 Observatoire pour la Conservation et l'Etude des Animaux et Milieux Marins (OCEAMM), 51 rue du Général de Gaulle, 59123, Zuydcoote, France; 4 Royal Belgian Institute of Natural Sciences (RBINS), Operational Directorate Natural Environment, 3^e^ en 23^ste^ Linieregimentsplein, B-8400, Oostende, Belgium; Institut National de la Recherche Agronomique, France

## Abstract

Bite-like skin lesions on harbour porpoises (*Phocoena phocoena*) have been suspected to be caused by grey seals (*Halichoerus grypus*), and a few field observations have been reported. Bite-like skin lesions observed on stranded animals were characterized by two main components: large flaps of loose or missing skin and blubber with frayed edges and puncture lesions. Definitive demonstration of predation by a grey seal was not reported so far in those stranded animals. In this study, five stranded porpoises with bite-like skin lesions were swabbed for genetic investigations. In addition, the head of a recently dead grey seal was used to mimic bite-like skin injuries on a porpoise carcass. Subsequently, the artificial skin injuries were swabbed, along with the gum of the seal used for inflicting them (positive controls). Total DNA was extracted from the swabs and was used to retrieve a fragment of mitochondrial DNA by PCR. Primers were designed to amplify a specific stretch of mitochondrial DNA known to differ between grey seals and porpoises. The amplicon targeted was successfully amplified from the positive control and from two of the stranded porpoises, and grey seal-specific mitochondrial DNA was retrieved from all those samples. We conclude that (1) it is possible to detect grey seal DNA from dead porpoises even after several days in seawater and (2) bite-like skin lesions found on dead porpoises definitively result from grey seals attacks. The attacks are most likely linked with predation although, in a number of cases, scavenging and aggressive behaviour cannot be excluded.

## Introduction

Skin lacerations and bites are frequently reported on stranded harbour porpoises (*Phocoena phocoena*), with a bite being defined as any rupture in the skin caused by the teeth of an animal [1]. The origin of lacerations can sometimes be associated with by-catch in fishing nets, while in other cases, the cause remains unclear. Finally, definitive distinction should be made between ante-mortem injuries and post-mortem depredation by scavengers (birds, dogs, red fox, wild boar,…), such post-mortem interference being well known in forensic pathology. Recently, bite-like skin lacerations on porpoises have been suspected to be associated with the predation by grey seals (*Halichoerus grypus*) [2]. Such lesions could be differentiated into two types: either large flaps of loose or missing skin or blubber with frayed edges or punctures. In both cases, lesions were considered as being ante-mortem and were highly similar to those inflicted by large dogs [1] even if excluded by Haelters *et al.*, 2012. Nevertheless, grey seal predation was not definitively demonstrated in those first reported cases. Recently, visual observations proved that grey seals were scavenging on, and attacking porpoises in France [3]. However, these latter observations were collected from a high distance and they did not definitively demonstrate that the grey seals were killing the porpoises [3]. In the absence of direct observation of predation, the genetic identification of predators is the gold-standard method in wildlife forensic pathology [4] and, in such cases, the method can be applied to definitively identify the predator. Indeed, in the forensic pathology perspective of this study, the objective was to demonstrate that bite-like skin lacerations were definitively inflicted by grey seals, and not by a dog or another potential predator in the case of a live stranded porpoise. Next to this, observations during the necropsy could indicate if the skin lacerations were inflicted on a live (predation or aggressive behaviour) or a dead porpoise (scavenging).

Here, we report on genetic evidence that grey seals are responsible for some bite-like ante-mortem skin injuries on harbour porpoises.

## Material and Methods

### Animals

Marine mammals, stranded on the Belgian and northern France coastline are investigated to determine their cause of death. The Department of Veterinary Pathology of the University of Liege is mandated officially by the Royal Belgian Institute of Natural Sciences and the French Stranding Network (UMS Pelagis, University of La Rochelle, France) to perform such post-mortem investigations. Five well preserved mutilated harbour porpoises (conservation code 2–3) with evidence of haemorrhagic ante-mortem skin injuries were selected, necropsied and sampled using a standard procedure [5]. For genetic investigations, bite-like injury lesions were swabbed and sampled, and subsequently frozen (−20°C). In addition, the head of a recently dead grey seal was used to mimic bite-like skin injuries on a porpoise carcass and the artificial skin injuries and the gum of the seal were swabbed (positive controls).

DNA was extracted from samples and swabs using Nucleospin Tissue kit (Macherey-Nagel, Germany), following the manufacturer's recommendations. Primers were designed based on the method proposed to discriminate grey seal and harbour seal (*Phoca vitulina*) DNA [6] with adaptations to maximize differences between seal and porpoise sequences. Using mitochondrial 16S ribosomal DNA sequences from GenBank for grey seal (GenBank Accession no. X72004) and harbour porpoise (GenBank Accession no. AJ554063.1), the designed primers amplified a region of the 16S gene, 249 bp for the grey seal (Seal 16S F: 5′-CAAGAATTTTAATGTAAGCTTAAAATATA; Seal 16S R: 5′-TCTTGTTACTCATATTAGCATTGTCT) and 224 bp for the porpoise (Porp 16S F: 5′-GCTTTTTAGAAACGGATACAACC; Porp16S R: 5′- GCGAGGAGAAAATCTTTCTTG). Amplification by PCR was performed in 25 µl, with ∼100 ng DNA, 0,5 µM of each primer and 12,5 µl PCR Mastermix (2x) (Thermo Scientific, USA). PCR reactions were performed on a Mastercycler Pro Thermocycler (Eppendorf, Germany) with the following parameters: 95°C for 2 min, then 40 cycles of 95°C for 20 sec, 43°C for 15 sec and 72°C for 45 sec, followed by 72°C for 2 min. Positive (grey seal) and negative controls (porpoise) were used.

PCR products were subjected to electrophoresis on 1% agarose gel (SYBR Safe DNA Gel Stain, Invitrogen, USA) and visualized via UV light. Subsequently, bands were cut and purified with the Nucleospin Gel and PCR Clean-Up kit (Macherey-Nagel, Germany). Purified products were cloned in pCRII-Topo plasmid (Invitrogen, USA) and sequenced with automated BigDye capillary sequencer ABI 3730 (Applied Biosystems, Life Technologies, USA), following the manufacturer's recommendations. NCBI GenBank Blast was used to confirm the species of amplified mitochondrial DNA fragments [7].

## Results

### Pathological investigations

**Figure 1:**
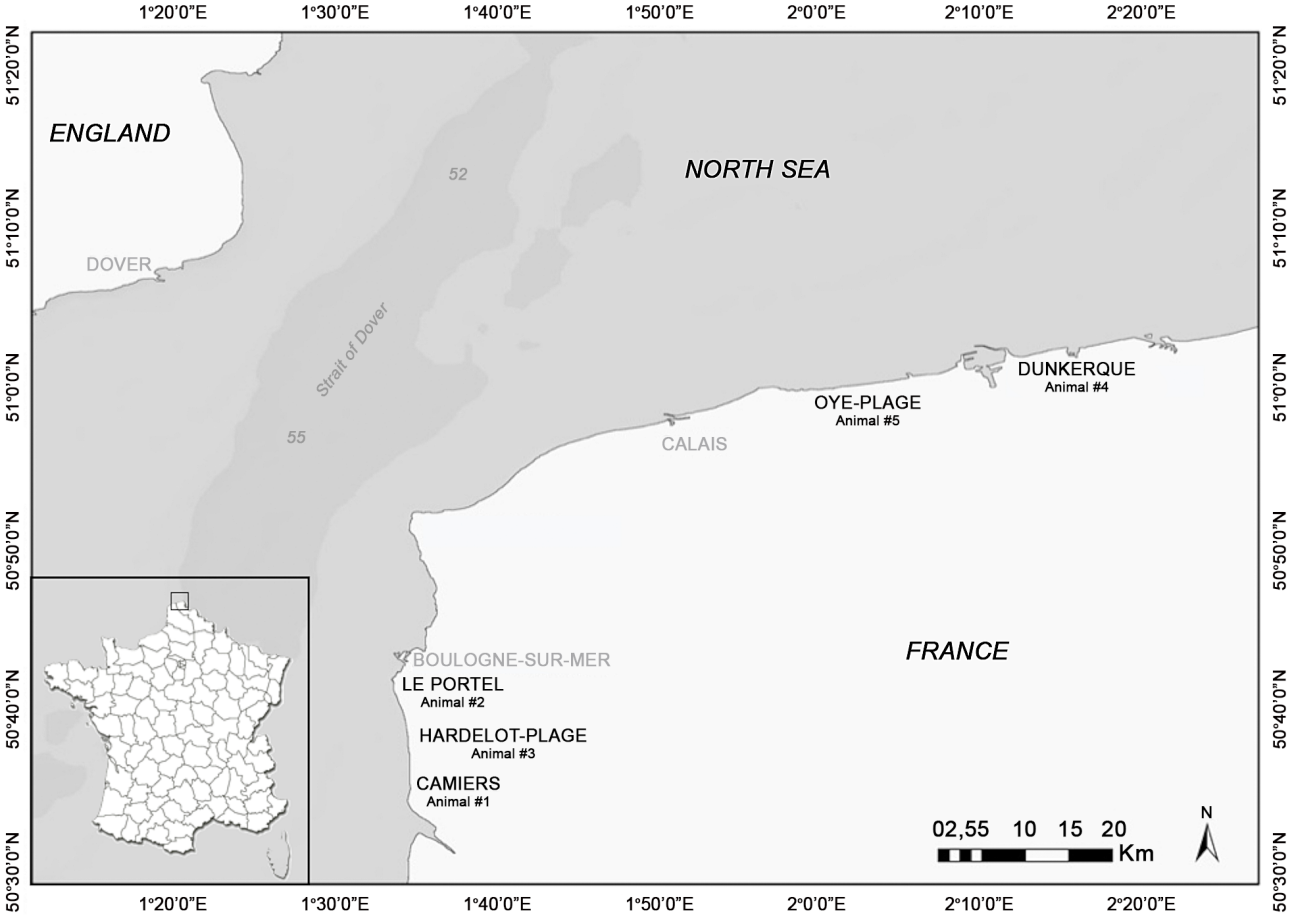
Geographical area where mutilated porpoises were collected.

**Figure 2:**
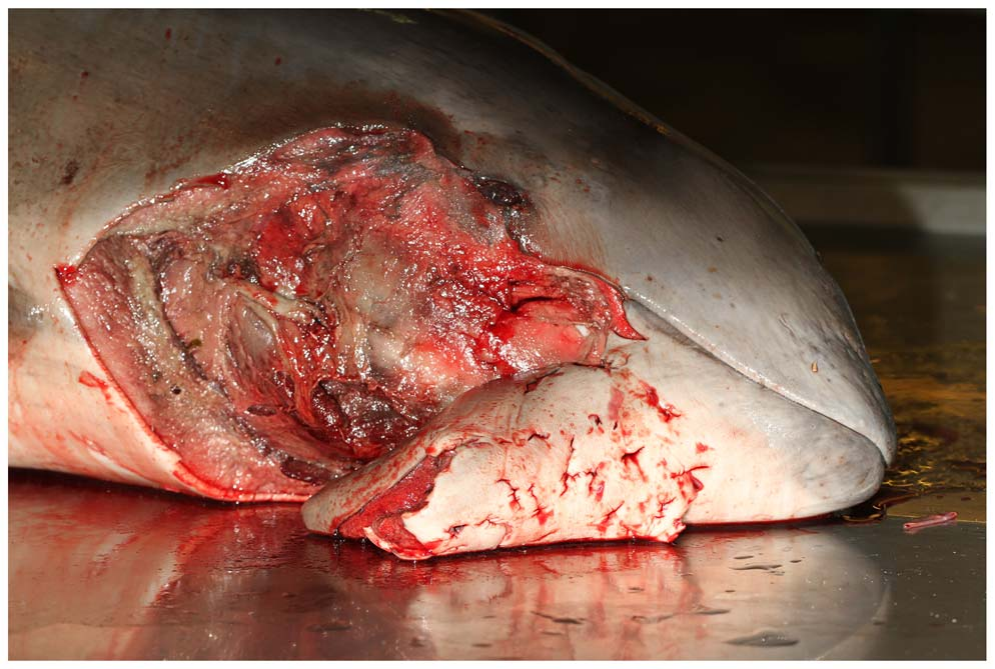
Skin punctures on the head of a porpoise.

The characteristics and post-mortem findings of the porpoises selected in the present study are summarized in Table 1. The five mutilated porpoises had stranded in winter (2012 and 2013) on the northern French coastline (Fig. 1). There were four juveniles: two females and two males (average body length: 113.5 cm, average body weight: 22.15 kg) and one adult female (161 cm and 49 kg). Four of the animals, including the adult female, were in a good nutritional state (medium blubber thickness: 21 mm). The external lesions found on those four porpoises (Animals #1, 2, 3 and 4) consisted of large skin and subcutaneous tissue lacerations or partly detached tissue flaps on the head, on the lateral and ventral side (animals #1, 2 and 3) or on the lateral side only (animal #4). The surrounding tissue was severely haemorrhagic suggesting an acute ante-mortem process. The edges of the lesions were straight, finely serrated and congestive except on animal #4, for which the edges were altered by scavengers' depredation. For this latter animal, clear marks were present on the beak and the flipper. In two cases (animals #1 and 2), skin punctures (Fig. 2) with haemorrhagic subcutaneous tissue were also present. In addition, those four porpoises showed evidence of recent feeding, severe pulmonary congestion and edema with abundant haemorrhagic froth in the airways and their cause of death was acute pulmonary edema, probably associated with a final asphyxia.

**Table 1:** Characteristics and relevant post-mortem investigations results of the five harbour porpoises.

	Porpoise #1	Porpoise #2	Porpoise #3	Porpoise #4	Porpoise #5
**Date of stranding**	29/02/13	28/01/13	21/01/12	25/11/13	24/02/13
**Place of stranding**	Camiers	Le Portel	Hardelot	Dunkerque	Oye plage
**Age**	Adult	Juvenile	Juvenile	Juvenile	Juvenile
**Body weight**	49 kg	19,5 kg	24.5 kg	23.6 kg	21 kg
**Bogy length**	161 cm	105 cm	110 cm	120 cm	119 cm
**Blubber thickness**	19 mm	23 mm	21 mm	21 mm	13 mm
**Skin lesion**	Lacerations, punctures	Lacerations, punctures	Lacerations	Lacerations, scavenging	Chronic lacerations
**Cause of death**	Pulmonary edema	Pulmonary edema	Pulmonary edema	Pulmonary edema	Acute pneumonia
**DNA results**	+	+	−	−	−

The fifth individual (animal #5) was severely emaciated (blubber thickness: 13 mm) and presented two parallel, linear, deep cutaneous lacerations, symmetric and bilateral on both size of the tailstock. The lacerations reached the subcutaneous tissue and showed thickened edges suggesting a chronic process. The edges of the lacerations were 4 cm apart on one side of the tailstock, and 5 cm on the other side. One large 8 cm abscess was present in the subcutis of the tailstock in the area of the lacerations. This last porpoise presented also 5 white parallel superficial scars limited to the epidermis on both sides of the tailstock. Moreover, this animal did not show recent feeding and presented a severe pulmonary parasitosis and an acute pneumonia. The cause of death was associated with an infectious process.

### Genetic analysis

**Figure 3:**
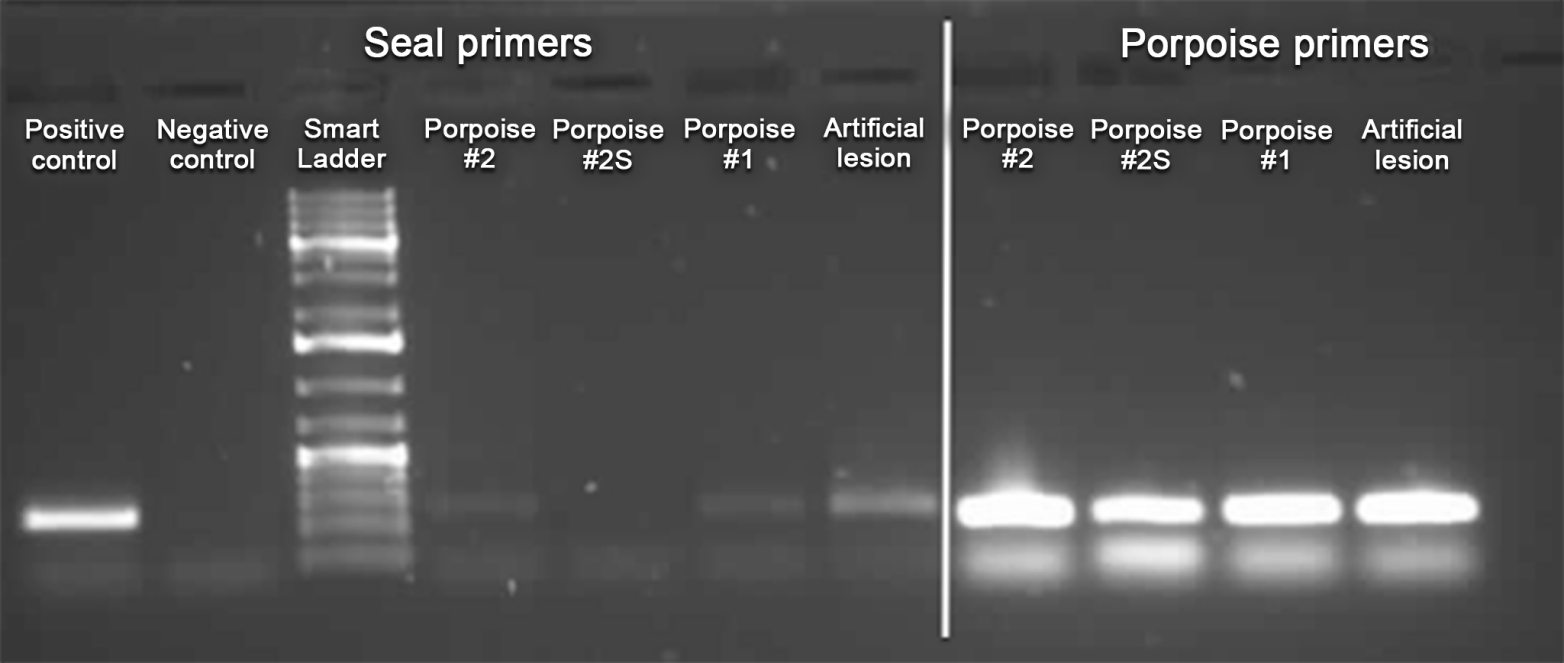
Typical post-PCR gel. Seal-specific primers, positive control: gum swab from a grey seal; negative control: skin swab from a harbour porpoise; porpoise #1 and #2: wound swabs, expected PCR product present (positive amplification). Artificial lesion: bite-like skin lesion deliberately inflicted post-mortem on intact porpoise with seal jaw. Porpoise-specific primers: all samples yielded expected PCR product.

The targeted mitochondrial DNA was successfully amplified from samples and swabs of the grey seal gum and the artificial skin lesion (positive controls). It was also amplified from two out of five tested porpoises (Fig. 3). The sequence of the PCR products is available on figshare (http://dx.doi.org/10.6084/m9.figshare.1146216). The comparison revealed that grey seal-specific mitochondrial DNA was retrieved from the gums and artificial skin injuries (positive controls) and also from the lesions of the two positive porpoises investigated. One was the adult female (animal #1), the other was a juvenile male (animal #2).

## Discussion

To date, two studies hypothesized that grey seals could be responsible for mutilations on harbour porpoises [2], [3]. Haelters *et al.* (2012) suggested that an adult bull grey seal would be the most likely suspected predator based on the distance between presumed canine teeth marks on the porpoises and the distance between the canine teeth in harbour and grey seal skulls [2]. Bouveroux *et al.* (2014) reported two direct observations of a grey seal attacking a porpoise and one observation of a grey seal scavenging on a dead porpoise – with no indication in the former case that the seal effectively had killed the porpoise. In the cases in which live porpoises were involved, it was not possible to definitely demonstrate that the seal was killing the porpoise, given the distance of the observation, while in the other case it was reported that the seal was removing strips of blubber and skin. In addition, in those cases the porpoises were not found stranded, and could not be necropsied. Then it was impossible to compare gross lesions with the previous study of Haelters *et al*, 2012, reinforcing the need of a genetic tool to identify definitively the animal species responsible for the bite-like skin lesions on stranded porpoises.

In the present study, similar bite-like skin lesions are reported and, additionally, grey seal-specific mitochondrial DNA was amplified and identified from the positive control and from the lesions of two ante-mortem mutilated porpoises out of the five tested. This study thus definitively confirms that grey seals are responsible for such mutilations and are thus most likely to have killed these porpoises. Indeed, the proposed cause of death for four of the porpoises was a severe lung edema, a typical lesion observed in case of asphyxia in cetaceans. For three of the porpoises (animals #1, 2 and 3), no significant lesions were observed other than the seal bites, suggesting that the seal attack was the most likely cause of the lung edema and the observations reported by Bouveroux *et al.* (2014) are consistent with this view. Indeed, those observations suggested that the grey seal attempted to submerge the porpoise, leading to the death resulting from asphyxia. As such, the associated hypoxia is responsible for the severe lung congestion and edema as described here. The location of the mutilations on the head further suggests that the grey seal attacks had focused on that area, possibly with the objective to sink and suffocate the porpoises. Grey seal DNA was amplified from two of these four porpoises, the two positive samples being collected deeply in bite-like puncture lesions. The two positively tested porpoises had no other lesions than those associated with the seal attack. Given that one was a healthy adult female, it seems that seals are not specifically selecting weak, sick or juvenile individuals.

For the two porpoises that tested negatively for the presence of grey seal DNA, puncture lesions were not present and the samples for genetic investigations were collected from the laceration edges. It can be expected that seal DNA disappears more easily on such laceration edges than in punctures. Among the two animals that had tested negatively, one was the porpoise stranded in Hardelot (Fig. 1) reported by Bouveroux *et al.* (2014), which had typical lesions of a seal predation. The other one had a large skin defect limited to the lateral side of the head and altered by scavengers (animal#4) but also showed the evidence of by-catch (net marks on beak and flippers), the capture probably resulting in the severe lung edema. The large flap defect could thus be independent of any seal predation. The negative PCR result reinforces these first conclusions. Further, the negative PCR result suggests that immersion of the porpoise's wound in sea water shared by both species and storage of a porpoise body close to other, seal DNA-positive porpoises does not result in accidental contamination.

The fifth porpoise (animal #5) presented a different pattern of lesions in terms of location and chronology. Indeed, the two parallel, symmetric and bilateral deep lacerations on both sides of the tailstock suggested that the porpoise had been bitten, the distance between the lacerations being compatible with the inter-canine teeth distance of a grey seal [2]. In addition, the five superficial and parallel scars suggest prints of seal claws. All those lesions were chronic and suggest that the porpoise survived the seal attack and died of another process. Nevertheless, an infectious process could have been the consequence of the attack since a large subcutis abscess was present in the area of the bites. Even if lesions on the tailstock are typical of a seal attack, the genetic investigations were negative for this porpoise. Different reasons could explain the negative result. As this animal survived for several days, it is possible that grey seal DNA was washed out of the lesion by seawater or by the inflammatory reaction. Another explanation is that only lacerations were present on this animal and not deep punctures.

The methodology of the detection of mitochondrial DNA is frequently applied to the identification of animal species [6], [8]–[10]. In marine ecosystems, the amplification of the 16S sequence, as in the present study, has allowed the discrimination between common and grey seal scat collected from haulouts [6]. Similarly, prey species identification using DNA from faeces or gut content has been used for diet analysis, the method being developed from scat samples of captive bottlenose dolphin (*Tursiops truncatus*) and then applied on wild bottlenose dolphins [9]. In terrestrial ecosystems, multiplex PCR targeting the mitochondrial cytochrome b gene has been used to allow detection of different species from degraded or trace samples [8]. More copies of mitochondrial DNA are found compared to nuclear DNA. Indeed there are two nuclear DNA copies per cell but numerous mitochondria per cell with 1 to 15 mitochondrial DNA copies. As such, the probability to detect mitochondrial DNA in trace or degraded samples such as scat, molted hair, processed meat and saliva traces is higher compared to nuclear DNA [8]. In addition, the degradation of mitochondrial DNA is slower given that it sits in an organelle protected by a protein coat [4]. Similar molecular tools were applied to analyse and successfully identify predator mitochondrial DNA in saliva swabbed from haemorrhagic wounds of caribou (*Ranifer tarandus*) calf in Newfoundland (Canada) [10]. In the study of Mumma *et al.* (2014), nuclear DNA microsatellite analysis had also allowed the identification of individuals and their sex. Mitochondrial DNA amplification is known to identify predators in terrestrial ecosystem, but it seems that the present study is the first to report such predator identification for the marine ecosystem.

The five porpoises with evidence of bite-like injuries had stranded in the northern part of France, more particularly in two areas: around Boulogne-sur-Mer (January 2012, January and February 2013), and around Dunkerque (February and November 2013). Bouveroux *et al.* (2014) reported predation and scavenging observations from the cliffs of Cap Gris-Nez (February and April 2013) and inside the harbour of Boulogne-sur-Mer (March 2013). The observations from Bouveroux *et al.* (2014) were made in the same area and during the same period as the cases reported in the present study. It is thus tempting to suggest that these attacks were performed by a limited number of adult grey seals or even by a single individual, but this has still to be demonstrated. The amplification of nuclear DNA microsatellites from bite skin lacerations of porpoises could be useful to identify the seal gender and even discriminate individual seals.

## Conclusions

We conclude that (1) it is possible to detect grey seal DNA from lesions in dead porpoises even after several days in seawater and post-stranding storage; (2) bite-like skin lesions found on dead porpoises in our context definitively result from grey seal attacks, likely linked with predation, although aggressive behaviour cannot be excluded; (3) systematic detailed necropsies should be performed on every stranded marine mammals as the external observation of skin lacerations can have different origins and should be combined with internal investigations; (4) as, in this study, grey seal DNA was only retrieved from acute and puncture lesions, sample collection in the context of predator species identification should focus on such lesions, and (5) amplification of nuclear DNA sequences would be useful to identify the sex of the predator or even the individual.
